# Randomized Polypill Crossover Trial in People Aged 50 and Over

**DOI:** 10.1371/journal.pone.0041297

**Published:** 2012-07-18

**Authors:** David S. Wald, Joan K. Morris, Nicholas J. Wald

**Affiliations:** Wolfson Institute of Preventive Medicine, Barts and the London School of Medicine and Dentistry, Queen Mary University of London, London, United Kingdom; University of British Columbia, Canada

## Abstract

**Background:**

A Polypill is proposed for the primary prevention of cardiovascular disease in people judged to be at risk on account of their age alone. Its efficacy in reducing cholesterol and blood pressure is uncertain.

**Methods:**

We conducted a randomized double-blind placebo-controlled crossover trial of a Polypill among individuals aged 50+ without a history of cardiovascular disease and compared the reductions with those predicted from published estimates of the effects of the individual drugs. Participants took the Polypill (amlodipine 2.5 mg, losartan 25 mg, hydrochlorothiazide 12.5 mg and simvastatin 40 mg) each evening for 12 weeks and a placebo each evening for 12 weeks in random sequence. The mean within-person differences in blood pressure and low density lipoprotein (LDL) cholesterol at the end of each 12 week period were determined.

**Results:**

84 out of 86 participants completed both treatment periods. The mean systolic blood pressure was reduced by 17.9 mmHg (95% CI, 15.7–20.1) on the Polypill, diastolic blood pressure by 9.8 mmHg (8.1–11.5), and LDL cholesterol by 1.4 mmol/L (1.2–1.6), reductions of 12%, 11%, and 39% respectively. The results were almost identical to those predicted; 18.4 mmHg, 9.7 mmHg, and 1.4 mmol/L respectively.

**Conclusion:**

The Polypill resulted in the predicted reductions in blood pressure and LDL cholesterol. Long term reductions of this magnitude would have a substantial effect in preventing heart attacks and strokes.

**Trial Registration:**

Controlled-Trials.com ISRCTN36672232

## Introduction

A combination pill for the prevention of cardiovascular disease was first described in 2000 [1], and called a Polypill. [2] Several Polypills have been made and assessed with respect to cholesterol and blood pressure reductions in people selected, on the basis of multiple risk factor measurement, to be at increased risk of cardiovascular disease. These trials[3]–[6] showed significant reductions in blood pressure and cholesterol, but estimates of the magnitude of the effects varied, and were lower than those expected from previous research. [2], [7], [8] There are several reasons for this. [9] The four previous Polypill trials underestimated efficacy because of poor adherence to treatment in the treated group or unscheduled treatment in the control group. In two trials the proportion of participants who stopped taking their treatment was 16% [3] and 23% [4]; in a third trial 30–35% were reported as not having taken their allocated pills [5]; in the fourth trial [6] more than 68% of controls took the Polypill components and the authors acknowledged that the trial could not estimate the risk factor reduction on the Polypill used. All four previous trials used a parallel group design in which participants who either stopped taking their treatment or took additional treatment needed to be included in an intention-to-treat analysis to avoid selection bias but at the cost of underestimating efficacy.

The uncertainty over the true efficacy prompted us to conduct a trial of a Polypill, intended for the primary prevention of cardiovascular disease in people selected only on the basis of age (50 years and over). This Polypill consisted of three blood pressure lowering agents (amlodipine 2.5 mg, losartan 25 mg and hydrochlorothiazide 12.5 mg), all at half standard doses and simvastatin 40 mg (standard dose). We used a randomized crossover design to avoid the under-estimation of the risk factor reduction that may have occurred in the previous trials.[3]–[6] The aim of the study was to quantify the effect of this Polypill on blood pressure and serum LDL cholesterol and to compare the observed effects with those previously predicted. [2], [7], [8].

## Methods

The protocol for this trial and supporting CONSORT checklist are available as supporting information; see Checklist S1 and Protocol S1.

### Ethics Statement

Ethics Committee approval was obtained from the North London Research Ethics Committee 1 and a clinical trials authorization obtained from the Medicines and Healthcare products Regulatory Agency. The trial is registered on the ISRCTN Register (ISRCTN36672232).

### Trial Design

We conducted a randomized placebo-controlled double-blind crossover trial of the Polypill described above, manufactured for the study group by Cipla, India. To help achieve high adherence during the trial, participants were recruited from individuals already taking simvastatin and blood pressure lowering drugs as part of a cardiovascular disease prevention programme. [10] Individuals were eligible to participate in the programme and, therefore, also the trial if they were aged 50 or over without an upper age limit, had no self reported history of cardiovascular disease, and were not taking drugs contraindicated with the Polypill components. Participants were not selected based on blood pressure or cholesterol measurements. Recruitment to the trial was limited to people who lived in London or could easily travel to London, where the trial was conducted. Written consent was obtained from the participants.

**Figure 1:**
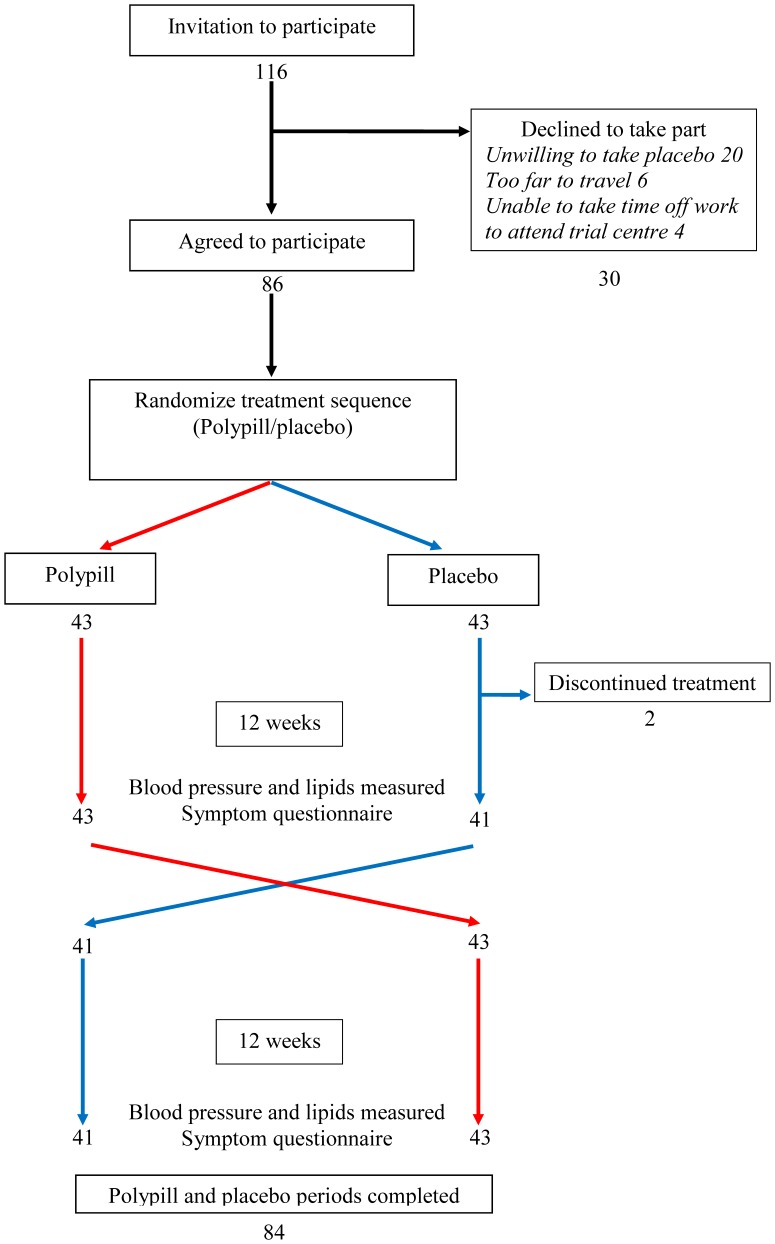
Flow chart of crossover trial.

Figure 1 is a flow chart of the trial. Trial participants took the Polypill (amlodipine 2.5 mg, losartan 25 mg and hydrochlorothiazide 12.5 mg) for 12 weeks, and an identical placebo for 12 weeks, the sequence being allocated at random. Neither the participants nor the investigators knew the sequence (ie. “double-blind”). Study treatment was taken once a day in the evening because of the greater efficacy of simvastatin when taken in the evening than in the morning. Existing blood pressure and cholesterol lowering treatments were discontinued at randomisation and no participant received additional blood pressure or cholesterol lowering treatment during the study. The randomization sequence was generated in advance by a computer random number generator, in blocks of 4. Date of birth, height, weight, and sex were recorded at randomization.

Blood pressure and lipid measurements were taken at the end of each 12 week period (usually between 10am and 2pm). Twelve weeks on the placebo was judged sufficient time for the drugs in the Polypill to have “washed out”, avoiding any carry-over effects because it has been shown that LDL cholesterol returns to pre-treatment levels within 4 weeks of stopping statin treatment [11] and it is standard practice to allow the same time period for blood pressure lowering treatment. [12] Blood pressure was measured, seated, after five minutes rest, with an automated sphygmomanometer (Omron, model 705IT, Kyoto, Japan); the average of two measurements, taken from the same arm, five minutes apart, was used. Venous blood samples were refrigerated, spun within 2 hours of collection and analysed within 6 hours in the Wolfson Institute of Preventive Medicine, London. Direct quantitative determinations of serum lipids were performed, using a Beckman Coulter Synchron analyser, to avoid the need for fasting.

The trial was not designed to assess side-effects of the Polypill but, as a safety measure, at the end of each 12-week treatment period participants completed a questionnaire on recognized side-effects of the component medications (cough, muscle ache or pain, ankle swelling, flushing, rash, tongue and lip swelling) and answered an open question on any other symptoms that might be attributable to the study medication. Adherence was assessed by a study investigator counting the remaining pills in each participant’s blister pack at the end of each treatment period. At the end of the trial participants continued on the Polypill.

### Statistical Methods

The main statistical analysis was a comparison of the mean within-person absolute and relative differences in systolic and diastolic blood pressure and the five lipid measurements (LDL cholesterol, HDL cholesterol, total cholesterol, triglyceride and ApoB) after taking the polypill compared with after taking the placebo. Paired t-tests were used to calculate statistical significance. Statistical power calculations indicated that 80 participants would provide a greater than 90% power with an alpha level of <0.05 (two tailed) if the LDL cholesterol reduction was at least 1.3 mmol/L (standard deviation 1 mmol/L) and diastolic blood pressure reduction was at least 9 mmHg (standard deviation 6 mmol/L).

The observed effects of the Polypill were compared with those expected from meta-analyses of the individual components. [1], [2], [7], [8] The cholesterol meta-analysis yielded the estimates that 40 mg simvastatin lowered LDL cholesterol by 37%, a relative reduction that was independent of the pre-treatment LDL cholesterol value, while the absolute cholesterol reduction (in mmol/L) increased with pre-treatment level. The blood pressure meta-analysis showed that the three blood pressure lowering drugs used in our Polypill would lower blood pressure by 19.9 mmHg systolic and 10.7 diastolic from pre-treatment values of 150 mmHg, and 90 mmHg, respectively, and published regression equations [13] were used to predict the reductions from lower pre-treatment blood pressure levels. Statistical analyses were performed using Stata (version 10, StataCorp LP, College Station, Texas).

## Results

Between December 2010 and March 2011, 116 individuals were invited to join the trial, 86 participants accepted and 84 completed both treatment periods (see figure 1). Participant characteristics are shown in table 1 including pre-trial medicines discontinued and blood pressure and lipid measurements at the end of the placebo period. Two participants did not complete the trial (both stopped during the placebo period), one because she wanted to stop treatment and the other because she wanted to be sure she received it. The sequence of treatment periods was balanced (43 Polypill first, and 43 placebo first). Table 2 shows the mean absolute and relative differences in blood pressure and lipid measurements while taking the Polypill compared with the placebo. Compared with placebo, mean systolic blood pressure reduced by 17.9 mmHg (12%) and diastolic blood pressure by 9.8 mmHg (11%) on the Polypill. LDL cholesterol reduced by 1.4 mmol/L (39%). In addition, apoB, total cholesterol, and triglyceride (even though non-fasting) levels were significantly reduced but HDL was not.

**Table 1:** Characteristics of the trial participants including medications discontinued at randomisation and blood pressure and lipid measurements at the end of the placebo period.

**Characteristic**	
Age: mean (range)	59 (51–77)
Sex	
Number of men (%)	64 (74)
Number of women (%)	22 (26)
Smoking	
Number of Smokers	8 (9)
Body Mass Index (kg/m^2^): mean ±150	28±4
**Pre-trial prescribed medicines discontinued at randomisation: number (%)**	
simvastatin (40mg/d)	86 (100)
bendrofluazide (1.25mg/d)	86 (100)
amlodipine (2.5mg/d)	86 (100)
lisinopril (5mg/d)	69 (80)
losartan (25mg/d)	17 (20)
**Blood Pressure at end of placebo period: mean** ±**1SD**	
Systolic (mmHg)	143±16
Diastolic (mmHg)	86±10
**Lipid measurements at end of placebo period: mean** ±**1SD**	
Total cholesterol (mmol/L*)	5.9±1.0
LDL cholesterol (mmol.L*)	3.7±0.9
HDL cholesterol (mmol/L*)	1.4±0.4
Triglycerides (mmol/L*)	1.8±1.0
ApoB (g/L)	1.2±0.2

* Conversion to mg/dL: multiply by 38.67 for cholesterol and by 88.57 for triglyceride.

**Table 2:** Absolute and relative difference in blood pressure (BP) and lipid measurements in 84 participants while taking the Polypill, compared with the placebo.

	Absolute difference*			Relative difference**		
	Mean	95% CI	P	Mean	95% CI	P
SystolicBP(mmHg)	−17.9	−20.1,−15.7	<0.001	12%	14%,11%	<0.001
DiastolicBP(mmHg)	−9.8	−11.5,−8.1	<0.001	11%	13%,9%	<0.001
LDLcholesterol(mmol/l)	−1.4	−1.6,−1.2	<0.001	39%	43%,34%	<0.001
ApoB(g/L)	−0.4	−0.4,−0.3	<0.001	30%	34%,26%	<0.001
Cholesterol(mmol/l)	−1.6	−1.8,−1.3	<0.001	27%	30%,23%	<0.001
Triglycerides(mmol/l)	−0.4	−0.6,−0.2	0.001	23%	33%,13%	0.001
HDLcholesterol(mmol/l)	0.03	−0.03,0.08	0.34	−2%	2%,−6%	0.34

* Polypill minus placebo.

** (Placebo minus Polypill)/placebo for each participant. Mean percentage differences are not precisely derivable from the absolute differences because of rounding and averaging within-person differences.

**Figure 2:**
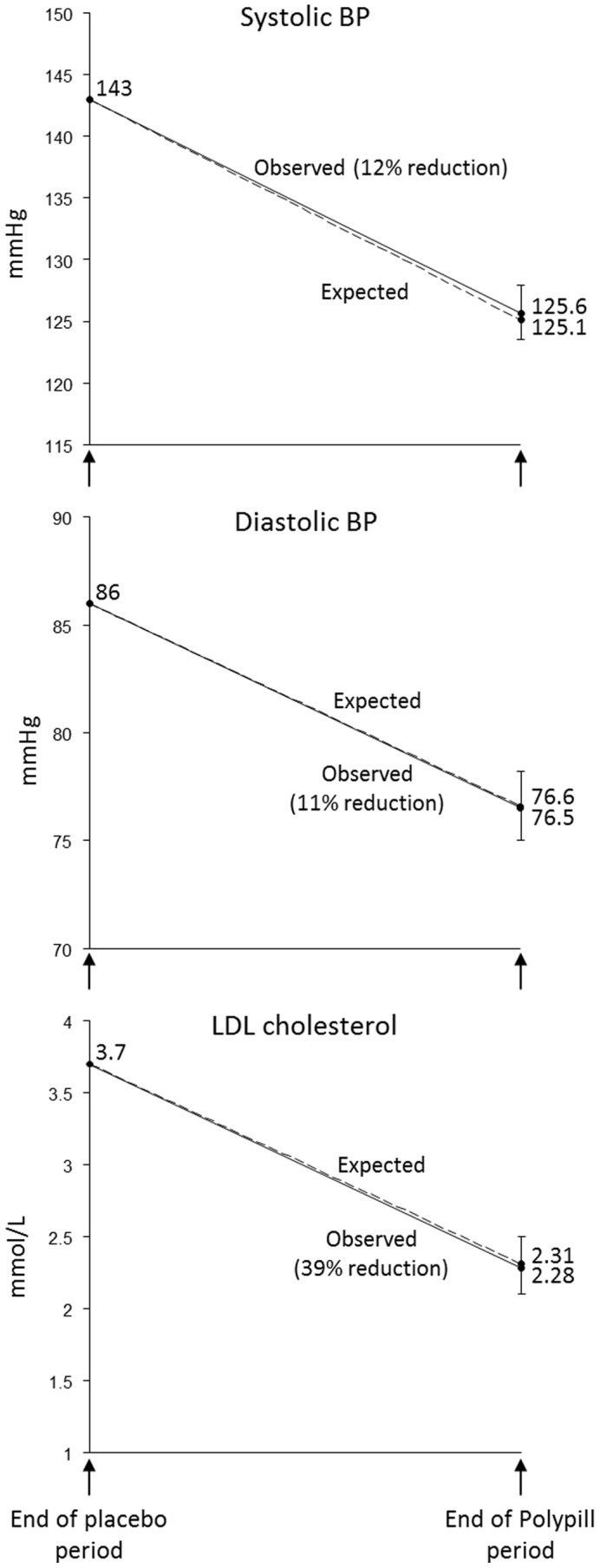
Observed and expected reduction in blood pressure and LDL cholesterol on the Polypill.

Figure 2 shows the observed blood pressure and LDL cholesterol reductions compared with those expected from meta-analyses of randomized trials of the individual classes of Polypill components [1], [2], [7], [8], allowing for the lower baseline blood pressure and LDL cholesterol in our trial participants (systolic blood pressure 143 mmHg compared with 150 mmHg, diastolic blood pressure 86 mmHg compared with 90 mmHg, and LDL cholesterol 3.7 mmol/L compared with 4.8 mmol/L). Figure 2 shows that the trial results using the Polypill are close to those expected.

Pill counts at the end of each treatment period indicated that 98% of participants (80/82, two forgot to bring their blister packs) took more than 85% of their allocated pills (same numbers on Polypill and placebo). Twenty four out of 84 participants reported one or more symptoms on the Polypill, compared with 11 on the placebo (p = 0.01) but none of these participants considered them troublesome enough to stop treatment. Muscle ache was more common on the Polypill than on placebo (9 v 1). No participant had a serious adverse event.

## Discussion

This is the first randomized trial of a Polypill offered to people on the basis of age alone; participants accepted that there was no need to know their blood pressure or their cholesterol level measurements which add little to age in predicting ischaemic heart disease events and strokes.[14] The results show substantial reductions in blood pressure and LDL cholesterol that were remarkably similar to those previously predicted.[1], [2], [7], [8] The results are likely to be more accurate than those from the other Polypill trials [3]–[6] because while the other trials used a parallel group design, we used a crossover design that has greater statistical power and avoids the dilution of the estimate of effect due to participants who did not take their allocated treatments being included in the results as part of the intention-to-treat analysis. Our results show that the original predictions were accurate and not overestimated as has been suggested.[3], [15].

Our trial has important strengths. The crossover design, with each participant being his or her own control, has the advantage of providing more precise and accurate estimates of blood pressure and cholesterol lowering effects with many fewer participants than would be needed using a parallel group design to achieve the same level of precision and, unlike a parallel group trial, participants who discontinue treatment can be excluded without introducing bias (two participants in our trial). A parallel group trial design would require four to five times as many participants to yield results with the same level of precision assuming 100% adherence to the allocated treatment, which is rarely achievable in practice with such a design. In our trial we achieved high adherence by recruiting participants already taking Polypill components. However, achieving a high adherence to treatment in this way means that the trial also has two limitations. First, the observed adherence rate cannot be used to estimate adherence in the general population. Second, the results on tolerability cannot be used to estimate the prevalence of side effects in people who have not previously taken Polypill components. In spite of this, the results revealed an increased prevalence of muscle ache associated with statin use that was not severe enough to stop treatment. Previous work has estimated side effect rates. In one study 7% of individuals did not tolerate one or more of the drugs used in our Polypill [10] (1.25 mg bendrofluazide used instead of 12.5 mg hydrochlorothiazide); about half of this 7% needed modification of the regimen (4%) and half (3%) stopped treatment.

The Polypill we used excluded folic acid and aspirin. Folic acid was excluded because obtaining regulatory approval for a Polypill that included folic acid was judged to be uncertain given the lack of proven efficacy in clinical trials. Aspirin was excluded because the risk of bleeding in people without existing cardiovascular disease might be unacceptable in relation to the expected benefit although recent work indicating a protective effect against cancer may alter this assessment.[16], [17].

Given the composition of the Polypill we used, and the observed LDL cholesterol and blood pressure reductions, the predicted effect in reducing ischaemic heart disease events is 72% and in reducing stroke is 64% based on evidence of the quantitative relationship between the two risk factors and risk.[2], [13], [18]–[22] This Polypill, designed principally for primary prevention, therefore has considerable potential for the prevention of cardiovascular disease.

## Supporting Information

Checklist S1 **CONSORT Checklist.**
(DOC)

Protocol S1 **Trial Protocol.**
(DOC)
