# Supplementary material for: Randomized Polypill Crossover Trial in People Aged 50 and Over
Source: PLoS One. 2012 Jul 18;7(7):e41297. doi: 10.1371/journal.pone.0041297 (PMC3399742; doi:10.1371/journal.pone.0041297)
Supplement: Protocol S1 — Trial Protocol. (DOC) [file pone.0041297.s002.doc]

**Polypill Prevention Trial I**

**Randomised controlled trial of the Polypill for multiple risk factor reduction**

**EudraCT number:** 2010-019874-34

**PROTOCOL**

Version 1.1 19 October 2010

Trial Centre

Wolfson Institute of Preventive Medicine

Queen Mary & Westfield College, University of London

(The Barts and The London

Queen Mary’s School of Medicine & Dentistry)

Charterhouse Square

London EC1M 6BQ

Representative of the Sponsor:

**Gerry Leonard**

**Head of Resources**

Joint R&D Office

5 Walden Street

London

E1 2EF

Phone: 020 7882 7260

Email: [gerry.leonard@bartsandthelondon.nhs.uk](mailto:gerry.leonard@bartsandthelondon.nhs.uk)

**Signature Page**

The clinical study as detailed within this research protocol **(Version 1, dated 28 June 2010)**, involves the use of an investigational medicinal product and will be conducted in accordance with the Royal College of Physicians Ethics principles, Research Governance Framework for Health & Social Care (2005), Principles of ICH-GCP, and the current regulatory requirements, as detailed in the Medicines for Human Use (Clinical Trials) Regulations 2004 (UK S.I. 2004/1031).

**Investigators: Signature Date**

Dr David Wald (PI)
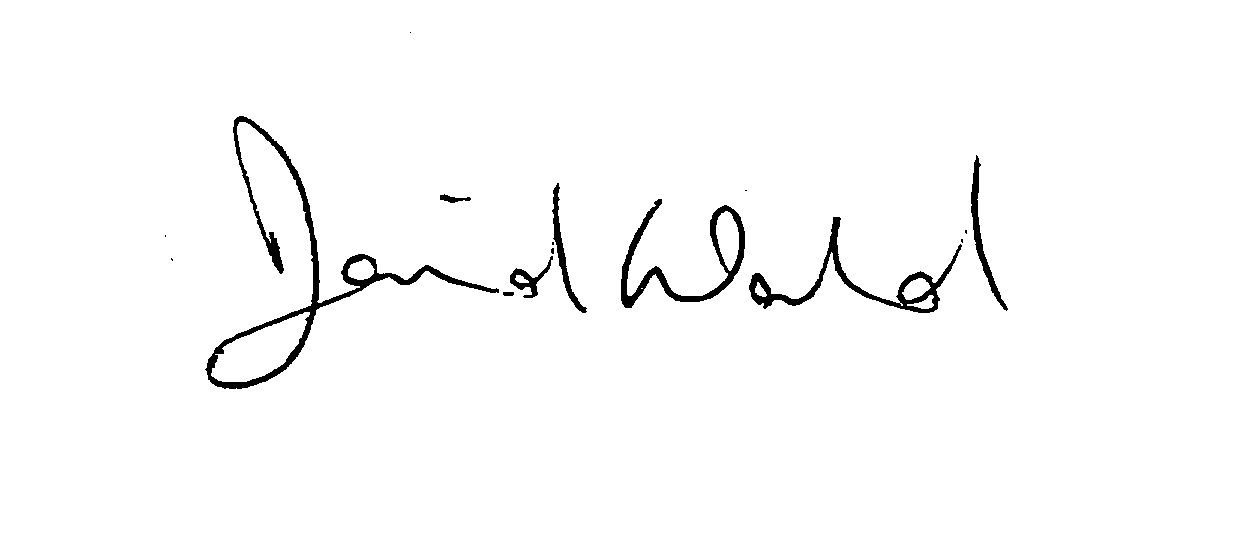
 28/06/10

Professor Joan Morris
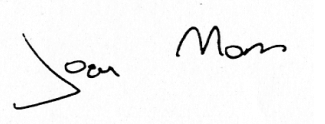
 28/06/10

Professor Sir Nicholas Wald
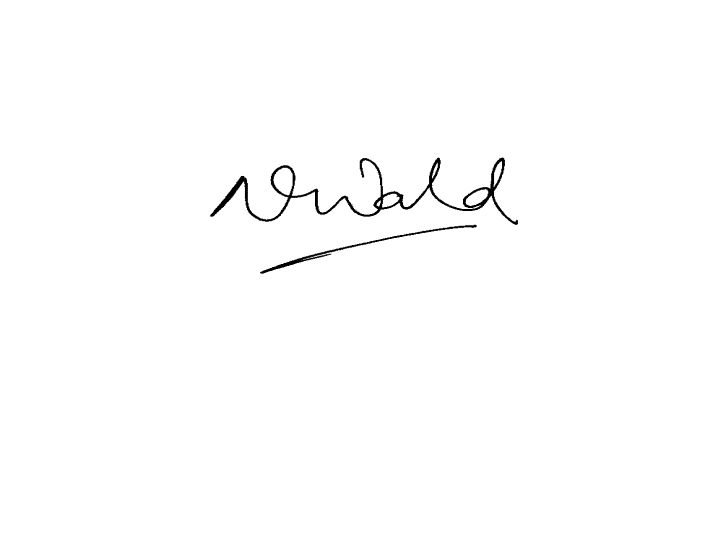
 28/06/10

**Site:** Wolfson Institute of Preventive Medicine, Barts and the London School of Medicine, Queen Mary & Westfield College, University of London

| **TITLE** | Polypill Prevention Trial 1 |
| --- | --- |
| **SHORT TITLE** | PPT1 |
| **Protocol Version Number and Date** | Version 1.1 [19 October 2010] |
| **Methodology** | Randomised placebo controlled double blind cross-over trial |
| **Study Duration** | 6 months followed by a 24 month open label monitoring period |
| **Study Centre** | **Wolfson Institute of Preventive Medicine** |
| **Objectives** | To determine the placebo controlled reduction in serum cholesterol and blood pressure using the Polypill and to assess the prevalence of adverse effects |
| **Phase of the Trial** | Phase III |
| **Number of Subjects/Patients** | 100 |
| **Main Inclusion Criteria** | Men and women aged 50 and over without a history of cardiovascular disease or contraindications to taking the Polypill |
| **Statistical Methodology and Analysis** | To estimate the mean (and 95% confidence intervals) for the within person, placebo-adjusted reductions in serum cholesterol and blood pressure. The trial has 90% power to identify statistically significant reductions in serum cholesterol of 1.5 mmol/L and a reduction of systolic blood pressure of 18mmHg (or 9mmHg diastolic) at a 0.05 level of statistical significance. |

# Glossary of Terms and Abbreviations

ABPI Association of the British Pharmaceutical Industry

AE Adverse Event

API Active Product Ingredient

AR Adverse Reaction

ASR Annual Safety Report

CI Chief Investigator

CRF Case Report Form

EUCTD European Clinical Trials Directive

EudraCT European Union Drug Regulating Authorities Clinical Trials

GCP Good Clinical Practice

GMP Good Manufacturing Practice

IB Investigator Brochure

IMP Investigational Medicinal Product

IMPD Investigational Medicinal Product Dossier
JRO Joint Research and Development Office

MA Marketing Authorisation

MHRA Medicines and Healthcare products Regulatory Agency

Main REC Main Research Ethics Committee

PI Principle Investigator

QA Quality Assurance

QC Quality Control

QP Qualified Person for release of trial drug

Participant An individual who takes part in a clinical trial

RCT Randomised Controlled Trial

REC Research Ethics Committee

SAE Serious Adverse Event

SAR Serious Adverse Reaction

SDV Source Document Verification

SmPC Summary of Product Characteristics

SOP Standard Operating Procedure

SSA Site Specific Assessment

SUSAR Suspected Unexpected Serious Adverse Reaction

TMG Trial Management Group

TSC Trial Steering Committee

**Index Page Number**

1. **Introduction 9**
   1. Abstract 9

1.2 Background 9

- 1. Investigational Medicinal Product (Polypill) 10
  2. Preclinical studies 11
  3. Clinical Studies 11
  4. Rationale and Risks/Benefits 12

**2.** **Study Aims and Design of Proposed Investigation 12**

2.1 Trial Objectives 12

2.2 Trial Design 12

2.3 Flow Diagram of Trial 13

**3. Study Population 14**

- 1. Inclusion Criteria 14
  2. Exclusion Criteria 15

**4. Investigational Medicinal Product 15**

4.1 IMP Supply 16

4.2 Preparation of IMP 16

4.3 Packaging and Labeling 16

4.4 Accountability/Receipt/Handling of IMP 17

4.5 Dispensing of IMP 17

4.6 Concomitant Therapies 18

4.7 Destruction of IMP 18

**5. Study Procedures 18**

- 1. Consent 18
  2. Data collection 18

5.3 Randomisation 18

5.4 Procedure for Unbinding 19

5.5 Clinical and Laboratory Assessments 19

5.6 Schedule of Assessment 20

**6. Study Endpoints 20**

6.1 Primary endpoint 20

6.2 Secondary endpoints 20

**7. Pharmacovigilance 20**

- 1. General Definitions 20

7.1.1 Adverse Event (AE)

7.1.2 Adverse Reaction (AR)

- - 1. Serious Adverse Event (SAE) or Serious Adverse Reaction (SAR)
    2. Suspected Unexpected Serious Adverse Reaction (SUSAR)

7.2 Investigators Assessment 21

7.2.1 Seriousness

7.2.2 Causality

7.2.3 Expectedness

7.2.4 Severity

7.3 Notification and reporting Adverse Events or Reactions 22

- 1. Notification and Reporting of Serious Adverse Events/SUSAR 22

7.4.1 All Serious Adverse Event (SAEs)

- - 1. Suspected Unexpected Serious Adverse Reactions (SUSARs)

7.5 Urgent Safety Measures 22

7.6 Annual Safety Reporting 23

7.7 Procedures for reporting blinded SUSARs 23

7.8 Overview of the Safety Reporting Process/Pharmacoviligance 23

responsibilities

**8. Statistical Considerations 24**

8.1 Number of Participants 24

8.2 Data Analysis 24

- 1. Data access and security 24

**9. Data Handling & Record Keeping 24**

9.1 Confidentiality 24

9.2 Case Report Form 25

9.3 Record Retention and Archiving 25

9.4 Compliance 25

9.5 Clinical Governance Issues - Ethical Considerations 25

9.6 Quality Control and Quality Assurance 26

**10. Trial Committees 26**

**11. Publication Policy 26**

**12. Insurance to Cover Indemnity for Non-Negligent Harm 26**

**13. References 27**

**Appendix A Sample Label 29**

**Appendix B Letter to invite participants of PPP 30**

**Appendix C Information Leaflet 31**

**Appendix D Consent form 35**

**Appendix E GP information letter 36**

**Appendix F Questionnaire 37**

**Appendix G Flow Chart 38**

**Appendix H ID Card 39**

**Appendix I Documentation of minor errors/corrections 40**

**1. INTRODUCTION**

***1.1 Abstract***

A randomised placebo controlled crossover trial of a four component Polypill (amlodopine, losartan, hydrochlorothiazide, simvastatin) will be conducted in 100 people who have not had a heart attack or stroke and are attending the Wolfson Institute of Preventive Medicine Polypill Prevention Programme, in which they are receiving blood pressure lowering drugs and a statin as separate pills. The aim of the trial is to estimate the effect of the new single Polypill Formulation on reducing serum cholesterol and blood pressure. Participants will be allocated in random sequence a regime of taking the Polypill for three months and taking a placebo for three months. Participants who complete the trial will continue to receive the Polypill for two years to monitor any reported adverse effects. Allowing for a 20% non-completion rate, the trial has 90% power to identify statistically significant reductions in serum cholesterol of 1.5 mmol/L and a reduction of systolic blood pressure of 18mmHg (or 9mmHg diastolic) at a 0.05 level of statistical significance.

***1.2 Background***

In 2003 Wald and Law with colleagues published a series of three papers proposing the “Polypill” strategy and showing that it would be expected to reduce a person’s risk of coronary heart disease (CHD) events and stroke by over 80%.1^-3^ The strategy is based upon using a combination of drugs in fixed doses in a single pill to reduce two cardiovascular risk factors simultaneously,1 and offering this to all people above an age (eg. 50 when heart attacks and strokes become common) without selection based on blood pressure or cholesterol levels.

The concepts underlying the strategy arise from the observations that:

1. For the cardiovascular risk factors, blood pressure and LDL cholesterol, the dose-response relationships with disease events show no evidence of a threshold below which a further reduction in the risk factor does not lead to further reduction in risk.^4-^ A reduction of specified magnitude in any of the risk factors produces a constant proportional reduction in risk irrespective of the pre-treatment value of the risk factor, 4 throughout the range of levels seen in Western populations. The evidence is based on meta-analyses of cohort (prospective observational) studies that show a continuous linear relationship between risk of CHD events or stroke (on a proportional scale) and each of the risk factors, with no threshold.5^-7^ This is supported by meta-analyses of trials of both blood pressure lowering drugs and cholesterol lowering drugs and CHD events and stroke, which show a similar proportional reduction in incidence irrespective of pre-treatment values 8^-11^ The Polypill is therefore appropriate for people at risk of CHD without selection based on measurement of risk factors.
2. The alternative means of preventing cardiovascular disease events by drugs, based on treatment of high blood pressure or high serum cholesterol, has limited efficacy. Heart attacks and strokes are an endemic problem because the average levels of risk factors in the population are raised and these different risk factors act synergistically.4 Offering preventive treatment to a small section of the population only (those with levels of one risk factor above an arbitrary threshold) will necessarily have limited effect. Lowering just one risk factor is also of limited effectiveness when it is the interaction between several that make cardiovascular disease common. A general intervention is needed that is simple, safe and well-tolerated.
3. The indication for taking the Polypill, for the prevention of first heart attacks and strokes is based upon the most discriminatory determinant of risk, namely age. Serum cholesterol, blood pressure and the other risk factors that can be changed are of great causal importance but are weak predictors of the diseases they cause. 4,2 They add little to risk estimation beyond age alone. Age 50 has been selected because 96% of deaths from coronary heart disease or stroke occur above this age in Britain so nearly all of these would be amenable to prevention using the Polypill.1

***1.3 Investigational Medicinal Product (Polypill)***

The Polypill is a single formulation, solid, orally administered tri-layered tablet combining four drugs that simultaneously reduce two separate cardiovascular risk factors (cholesterol and blood pressure). The Polypill has been specifically formulated to reduce the risk of cardiovascular disease by an estimated 75% with a very low incidence of adverse effects.The four component Polypill formulation is:

| ***Polypill Component*** | ***Dose*** | ***Class*** | ***Action on risk factor*** |
| --- | --- | --- | --- |
| Simvastatin 40mg | Standard | statin | LDL cholesterol |
| Hydrochlorothiazide 12.5mg  Amlodopine 2.5mg | Half-standard  Half-standard | thiazide  calcium-channel blocker | blood pressure lowering drugs |
| Losartan 25mg | Half-standard | angiotensin-II receptor antagonist |

***1.4 Preclinical Studies***

The individual components of the Polypill have been the subject of extensive pharmacokinetic and pharmcodynamic study (Summary of Product Characteristics are available on the APBI website http://www.abpi.org.uk/default.asp) and are all suitable for once daily dosing. The Polypill, manufactured by CIPLA (India) has met standard stability and dissolution requirements and produced satisfactory results in preliminary pharmacokinetic studies in 18 adult male subjects (aged 18-45). There were no adverse effects, no interactions between the specified components and the results demonstrated satisfactory preliminary bioequivalence in the rate and extent of absorption between the Polypill and its individual components taken separately.

***1.5 Clinical Studies***

The component drugs within the Polypill are all in established use and widely prescribed in medical practice.

*The blood pressure lowering drugs*

A meta-analysis of randomised placebo-controlled trials of blood pressure lowering drugs has shown that they are best used in low dose combinations. In general using such a drug at half standard dose substantially reduces the prevalence of adverse effects (such as lethargy, flushing or dizziness) with little (20%) loss of efficacy.3 Using the three different classes of drugs at half standard dose lowers blood pressure by an estimated 20 mmHg systolic and 11 mmHg diastolic, equivalent to a 46% reduction in risk of CHD events and a 63% reduction in stroke.3 This has a greater efficacy, with fewer adverse effects, than using one or two drugs at standard dose.

*LDL cholesterol reduction using simvastatin*

Randomised trials of statins and serum cholesterol reduction show that 40mg simvastatin daily lowers LDL cholesterol by an estimated 37% (about 1.8 mmol/L), with a prevalence of adverse effects (treated minus placebo) below 1%.2 The LDL cholesterol reduction of 1.8 mmol/L is equivalent to an estimated 61% reduction in risk of CHD events and a 17% reduction in stroke in the long term, and while little risk reduction is observed in the first year of treatment the maximal risk reduction is largely attained by the third year.2^,11^

Aspirin is not included in the formulation for healthy people because of concern that harm from bleeding episodes may outweigh efficacy in people without occlusive vascular disease (given that they are already receiving protection from the other Polypill components). ^13^

***1.6 Rationale and Risks/Benefits***

The Polypill holds the possibility of having a major impact in the prevention of cardiovascular disease, reducing the leading cause of death in both economically developed and economically developing countries. It would reduce the incidence of CHD events by an estimated 79% and stroke by 69% while causing minimal serious adverse reactions in less than 1 per 100,000 people per year arising from continued use of a statin in the face of muscle pain and weakness. The problem is reversible on withdrawal of treatment and can usually be completely avoided if treatment is discontinued when symptoms are reported. Minor adverse (such as flushing and dizziness) are expected in no more than about 10% and are reversible on stopping treatment.

The component drugs are established, readily available and all have been widely used in clinical medicine. There is substantial information on efficacy and safety for all of them. There have been a large number of randomised trials of each component drug, years of clinical use with post marketing surveillance, and no indication of significant unrecognised serious or life-threatening adverse effects. The drugs are commonly used in combination in clinical medicine, and there have been no suggestions of adverse effects with combinations of drugs that were not apparent with monotherapy. The current study is designed to directly estimate the serum cholesterol and blood pressure reductions using the Polypill and providing additional information on tolerability and safety.

The Polypill, if administered in the manner described in this document, offers a simple, highly effective and safe method of reducing cardiovascular disease in the community and has the potential of achieving substantial public health benefits.

**2. STUDY AIMS AND DESIGN OF PROPOSED INVESTIGATION**

***2.1 Trial Objectives***

**Primary Objective**: to determine the reduction in serum cholesterol and blood pressure using the Polypill.

**Secondary Objective**: to assess the prevalence of adverse effects.

***2.2 Trial Design***

The trial will adopt a double blind cross-over design in 100 subjects with three months on the Polypill and 3 months on placebo in random order.

***2.3 Flow Diagram of Trial
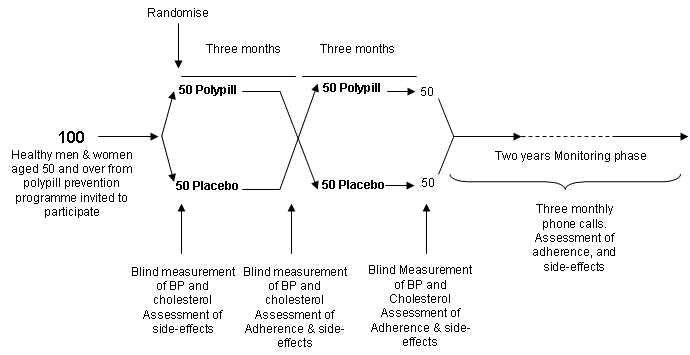
***

**Cross-over phase**

The Polypill or an identical placebo would be taken once a day (in the evening because simvastatin is more effective when taken in the evening than the morning). A cross-over design provides specific advantages over a parallel group design:

- A cross-over design offers the statistically most powerful method of determining the effect of the Polypill on risk factor modification (the cross-over design ensuring that each person is his/her own control).
- the cross-over design avoids dilution of results due to withdrawal/non-compliance since any participant who does not complete both treatment periods can be excluded without introducing selection bias.

The 3 month period on treatment (Polypill or placebo) and the recording of serum cholesterol and blood pressure at the end of that period ensure that any carry over effect of treatment in the preceding period will have washed out. The trial design and sample size allows for a 20% non-completion rate.

**Monitoring Phase**

At the end of the trial participants who have completed both cross-over phases will continue to receive the Polypill on a named patient basis (open label) for a period of monitoring (2 years) of adverse effects.

Every 12 weeks, during the 2-year monitoring phase, each participant will be contacted by phone or email to assess (i) adherence on the Polypill and (ii) whether any new symptoms have developed that may be side-effects of treatment.

(i) adherence will be assessed by asking whether there were any days in the last 4 weeks when the Polypill was not taken and if so for how many days.

(ii) side-effects will be assessed by questionnaire (see Appendix F)

During the two year monitoring phase a telephone helpline will be available for participants to use at any time to report any symptoms that may be side-effects of treatment or to ask any questions relating to their participation in the trial.

Pharmacovigilence will be undertaken in both the 6 month cross-over and 2 year monitoring phases of the study as specified in section 7 (7.1-7.8).

**3. STUDY POPULATION**

Participants will be recruited from the Wolfson Institute of Preventive Medicine Polypill Prevention Programme, a heart disease and stroke prevention service which adopts the polypill approach to cardiovascular disease prevention, using separate pills for each component, and includes men and women aged 50 and over without a history of heart attack or stroke. Participants of this programme will be sent an information leaflet and letter inviting them to participate 2 to 3 weeks ahead of them being telephoned by a researcher from the Wolfson Institute of Preventive Medicine to see if they are interested in taking part in the trial. If they are, a convenient time will be arranged for them to meet a study investigator or research assistant to discuss the study and answer any questions they may have.

***3.1 Inclusion Criteria***

Participants will be men and women aged 50 and over without a history of cardiovascular disease who have been taking cholesterol and blood pressure lowering drugs prophylactically, (ie. for the prevention of cardiovascular disease rather than its treatment) without selection on the basis of other cardiovascular risk factors and for whom there is no medical contra-indication to taking the Polypill (see 3.2 (ii) below). Thus entry to the trial will not be determined by blood pressure or cholesterol levels and so off-treatment (baseline) blood pressure and cholesterol will not be determined. Participants will have been taking individual blood pressure and cholesterol lowering drugs for at least 1 month prior to inclusion and will stop taking these during the study.

***3.2 Exclusion Criteria***

Exclusions include (i) a clinical history of cardiovascular disease (ii) Contraindications, as specified in the Summary of Product Characteristics, in respect of the individual component drugs (iii) any illness judged to contra-indicate participation in the trial (iv) pregnant or breast feeding women (becoming pregnant in the trial is unlikely since participants will be ≥50 years. Premenopausal women will be advised to use a method of contraception). (v) taking other specified drugs: lithium, cyclosporine or other calcineurin inhibitors, erythromycin or other macrolides, azole antifungals, ritonavir or other protease inhibitors, amiodarone.

**4. INVESTIGATIONAL MEDICINAL PRODUCT**

The Polypill has been formulated in a stable, solid dosage form containing 40mg simvastatin, 25mg losartan, 12.5mg hydrochlorothiazide and 2.5mg amlodipine besilate.

Simvastatin needs protecting from oxidation with an antioxidant under acidic conditions. The remaining actives are stable under a more neutral environment. To overcome this problem the Polypill has been formulated as a trilayered tablet:

Layer 1 (peach-colored) contains simvastatin 40mg

Layer 2 (white) contains Losartan Potassium 25 mg and Hydrochlorothiazide 12.5mg

Layer 3 (yellow) contains Amlodipine Besilate 25mg.

**
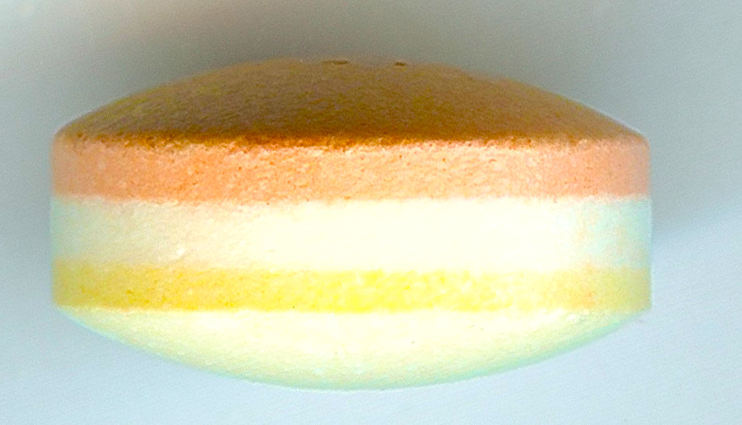
**

Amlodipine

Losartan & hydrochlorthiazide

Simvastatin

The Polypill has been manufactured by CIPLA, India at an MHRA GMP certified facility. The APIs (the individual components) and the Polypill all have Manufacturing Authorisations confirming GMP Certification:

| **Name of the API** | **Manufacturer** | **GMP compliance by** |
| --- | --- | --- |
| Amlodipine Besilate | Cipla Ltd., Bangalore, India | TGA, WHO & US-FDA |
| Hydrochlorothiazide | IPCA Lab. Ltd, India | WHO |
| Losartan potassium | Cipla Kurkumbh, India | TGA, WHO, US-FDA |
| Simvastatin | Biocon Ltd, India | EU-GMP of German authority |
| Polypill | Cipla Ltd., Patalganga, India | MHRA |
| Placebo | Cipla Ltd, Patalganga, India | MHRA |

The QP (Bilcare) will oversee the importation of the Polypill and placebo.

***4.1 IMP Supply***

Since the Polypill is manufactured outside the EU, importation, supply and release will be carried out by a QP (Pharmacist) working for an MHRA authorised Global Clinical Supplies Company (Blicare, MHRA site number 28707), including any required audits of the manufacturing sites in India. Use of the IMP in the proposed clincial trial will not commence until the necessary approvals have been received from the UK authority, the MHRA.

***4.2 Preparation of IMP***

Bilcare and the QP (Pharmacist) will be responsible for receiving the Polypill and placebo from CIPLA, inventory management, carrying out ID Testing (to confirm Polypill and placebo identities) and storing the Polypill and placebo under controlled and monitored conditions prior to QP Release for packaging, labelling and courier transport to the trial site.

***4.3 Packaging and Labeling***

The Polypill and placebo will be packaged and labelled by Bilcare in accordance with Annex 13 (Manufacture of Investigational Medicinal Products). Bilcare will be responsible for counting 100 tablets (10 blister packs each containing 10 tablets) in to 204 containers/boxes, applying a randomised label to each container, placing 2 containers in to a patient kit carton (one containing the Polypill and the other Placebo, labelled 1 or 2 in random sequence (see 5.3)) and applying a randomised label to produce 100 patient kits for use in the cross over study. A sample label is given in Appendix A. Bilcare will also count 100 Polypill tablets (10 blister packs each containing 10 tablets) in to 801 containers and apply a named patient label for use in the 2 year monitoring period. Storage will be at room temperature (15-25^o^C).

***4.4 Accountability/Receipt/Handling of IMP***

Bilcare will be responsible for receiving the IMP (Polypill and placebo tablets) from CIPLA, producing an inventory, inspecting the IMP, undertaking ID Testing as required and storing at 15-25^o^C under controlled and monitored conditions. Following QP Release the Polypill and placebo for the cross-over study will be dispatched to the Wolfson Institute of Preventive Medicine, Barts and the London School of Medicine, where the sealed 100 patient cartons (each containing a container of Polypill and a container of placebo) will be stored in a secure laboratory under controlled and monitiored conditions (temperature 15-25^o^C). The Wolfson Institute of Preventive Medicine has 3 levels of security; the campus is protected by 24 hour manned security and swipe card secure access, the building is protected by Wolfson Institute specific electronic swipe card access and the laboratory is staffed from 8am until 6pm and locked at all other times. Access to the IMP would be restricted to only the investigators of the study and IMP will be brought by the study investigators to the clinic in the Wolfson Institute of Preventive Medicine on the day participants are enrolled in the trial or reviewed. An inventory will be kept in the secure store to maintain an accurate record of all cartons removed from or returned to the secure store, the time and date they were removed or returned and by whom.

***4.5 Dispensing of IMP***

Each participant enrolled in the trial will be allocated a carton (each containing the container of Polypills and a container of placebo) in the order in which they are recruited (1 to 100). The number on the carton will be their unique study number. The Polypill or placebo will be dispensed by a medical doctor after seeing the participant in clinic within the Wolfson Institute of Preventive Medicine. The name of the participant, his or her date of birth and unique study number will be recorded in a dispensing log, together with the name of the dispensing doctor and the date and time the IMP is dispensed. Each container will have a peel-off label corresponding to the label on the container. This will be stuck in to the log against the time and date the IMP was dispensed. The participant’s initials and date of enrolment in the trial will also be written onto the carton which will be returned to the secure store following the visit. Any unused cartons will be returned to the secure store as explained in 4.4.

***4.6 Concomitant Therapies***

If any of the following drugs are required for other medical reasons during the trial then participants will discontinue participation in the trial: lithium, cyclosporine or other calcineurin inhibitors, erythromycin or other macrolides, azole antifungals, ritonavir or other protease inhibitors, amiodarone.

***4.7 Destruction of IMP***

Any unused IMP at the end of the study and any extensions if appropriate will be destroyed on site at the Wolfson Institute of Preventive Medicine, in accordance with standard procedures. This involves enclosure within a biohazard incineration bag with written instructions to burn without opening, sealed and sent for incineration.

**5. STUDY PROCEDURES**

***5.1 Consent***

All participants will be given an information leaflet with details of the study 2 weeks prior to enrolment and will be asked to give written consent prior to participation. Consent will also be obtained for blood sample storage for future research.

***5.2. Data Collection***

When a person agrees to participate the following will be recorded: Participant’s name, address, date of birth and GP details. Each participant will be allocated a unique trial number.

Details of the participant’s medical history will be recorded including, the presence of cardiovascular risk factors, whether they have diabetes, their smoking status and whether there are any other significant illnesses or contraindications to the Polypill.

***5.3 Randomisation***

Participants will be randomly allocated to receive either the Polypill during the first 3 month period and the placebo during the second three month period, or the placebo during the first 3 month period and the Polypill during the second three month period. A random sequence will be generated by computer in blocks of 12 to ensure balanced 1:1 randomisation. This sequence will be used by Bilcare to package and label the IMP cartons so that investigator and participant are blinded to the sequence.

***5.4 Procedure for Unblinding***

Code breaks will be kept securely by Bilcare and the Study Statistician for use at the end of the trial or during the trial if any clinical event arises that requires unblinding to be undertaken during the course of the trial. Any unblinding during the trial will take place in consultation with the study investigators (DW and NW) and be implemented through the study statistician who will hold the treatment allocation key. An internal rota will be arranged at the Wolfson Institute of Preventive Medicine to ensure, that out of hours, a responsible person will have access to the randomisation key.

***5.5 Clinical and Laboratory Assessments***

Blood pressure measurements will be made and blood collected during the initial cross-over phase and subsequent monitoring phase for statistical analysis by Wolfson Institute staff.

**Blood Pressure**

Blood pressure will be measured using an electronic sphygmomanometer and an appropriately sized cuff in the right arm of seated participants. Three readings will be taken and the mean calculated. Each participant’s blood pressure will be digitally recorded and stored, uniquely identifiable by the time and date when the measurement was made and the participants unique identifier number.

**Blood samples**

A venous blood sample, identifiable by the participants unique identifier number and the time and date when the sample was taken, will be collected and spun to provide serum for analysis of total cholesterol, HDL cholesterol, triglycerides and calculated LDL cholesterol.

**Other measurements**

A questionnaire will be administered at the start of the study to record the prevalence of symptoms commonly attributed to adverse effects of the Polypill components (eg, ankle swell, muscle ache) and at each subsequent visit to determine whether there have been any change in symptoms that could be attributed to adverse effects of treatment.

A questionnaire on adherence and a count of remaining tablets will be undertaken at 2^nd^ and 3^rd^ visits.

**Samples stored for future research**

Whole blood and serum will be collected at each visit and will be kept for future analyses at the Wolfson Institute laboratory. Whilst not all analyses can be anticipated at present, these might, for example, include studies to help identify why some people experience adverse effects form the components of the Polypill.

***5.6 Schedule of Assessment***

| Assessment | At Randomisation | Visit 1 (time=3 months) | Visit 2 (time =6 months) |
| --- | --- | --- | --- |
| History | x | x | x |
| Weight | x | x | x |
| Height | x |  |  |
| Blood Pressure | x | x | x |
| Venous blood | x | x | x |
| Questionnaire on symptoms | x | x | x |
| Pill counts of remaining tablets | - | x | x |

**6.** **STUDY ENDPOINTS**

***6.1 Primary end-point***

The primary endpoint is the placebo – adjusted reduction in total and LDL cholesterol and systolic and diastolic blood pressure.

***6.2 Secondary endpoints***

(i) the prevalence of adverse effects of treatment (treated minus placebo);

(ii) adherence to treatment

**7 PHARMACOVIGILANCE**

- 1. ***General Definitions***

*7.1.1 Adverse Event (AE)*

An AE is any untoward medical occurrence in a subject to whom a medicinal product has been administered, including occurrences which are not necessarily caused by or related to that product. An AE can therefore be any unfavourable and unintended sign, symptom or disease temporarily associated with the use of an Investigational Medicinal Product (IMP), whether or not considered related to the IMP. These will be recorded.

*7.1.2 Adverse Reaction (AR)*

An AR is any untoward and unintended response in a subject to an Investigational Medicinal Product (IMP). All adverse events judged by the reporting investigator as having a reasonable causal relationship to the IMP will be recorded.

*7.1.3 Serious Adverse Event (SAE) or Serious Adverse Reaction (SAR)*

An SAE is a fatal or life-threatening event resulting in hospitalisation or significant disability. Any SAE will be recorded.

Serious Adverse Reaction (SAR)

An SAR is an adverse reaction that is classed as serious and which is consistent with the information about the IMP as set out in the Investigator’s Brochure (IB).

- - 1. *Suspected Unexpected Serious Adverse Reaction (SUSAR)*

The definition of a SUSAR is any serious adverse event related to an IMP that is both suspected to be related to the IMP and unexpected. In this case the event is not outlined in the Summary of Product Characteristics (SmPC) or Investigator’s Brochure (IB) for that product.

***7.2 Investigators Assessment***

*7.2.1 Seriousness*

The Chief/Principal Investigator responsible for the participant, or in his absence an authorised medic within the research team will be responsible for assessing whether the event is serious according to the definitions given in section 7.1.

*7.2.2 Causality*

The Investigator will assess the causality of all serious adverse events/reactions in relation to the trial treatment according to the definition given. If the SAE is assessed as having a reasonable causal relationship, then it will be an SAR.

*7.2.3 Expectedness*

The investigator will assess the expectedness of all SARs according to the definition given. If the SAR is unexpected, then it is a SUSAR.

*7.2.4 Severity*

The Investigator will assess the severity of the event according to the following terms and assessments:

**Mild**: Some discomfort noted but without disruption of daily life

**Moderate**: Discomfort enough to affect/reduce normal activity

**Severe**: Complete inability to perform daily activities and lead a normal life

***7.3 Notification and reporting Adverse Events or Reactions***

## If the AE is not defined as SERIOUS, the AE will be recorded in the study file and the participant followed up by the research team. The AE will be documented in the participants’ medical notes (where appropriate) and the CRF.

***7.4 Notification and Reporting of Serious Adverse Events/SUSAR***

7.4.1 All Serious Adverse Event (SAEs) will be recorded in the subjects’ notes, the CRF, the sponsor SAE form and reported to the Joint Research and Development Office (JRO) within 24 hours of the PI or co-investigators becoming aware of the event. Nominated co-investigators will be authorised to sign the SAE forms in the absence of the PI.

7.4.2 Suspected Unexpected Serious Adverse Reactions (SUSARs) that occur during the trial will be reported to the JRO within 24 hours of the PI or co-investigator becoming aware of the event.

***7.5 Urgent Safety Measures***

The PI will inform both the MHRA and Main Ethics Committee **in writing within 3 days**, of any urgent safety measures he feels are required to ensure the safety and protection of the participants in the trial. The sponsor (JRO) will be sent a copy of the correspondence with regards to this matter.

***7.6 Annual Safety Reporting***

The Annual Safety Reports (ASR) will be sent by the PI to the sponsor, the MREC and MHRA (the date of the anniversary is the date on the “notice of acceptance letter” from the MHRA) using the ASR form. The CI will carry out a risk benefit analysis of the IMPs encompassing all events having arisen on the trial.

The PI will send the Annual Progress Report to the main REC using the NRES template (the anniversary date is the date on the MREC “favourable opinion” letter from the MREC) and to the sponsor.

***7.7 Procedures for reporting blinded SUSARs***

In the case of a blinded study, the treatment code for the patient will be broken in the reporting of a SUSAR. However, the blind should be maintained, where possible and appropriate, for staff that are involved in data analysis and interpretation. It is the allocated responsibility of the PI by the sponsor for pharmacoviligance management and reporting. In this instance, an allocated unblinded individual (s), with no involvement in data management of the study should be responsible for the unblinding event. The unblinding of single cases by the PI in the course of a clinical trial will only be performed if necessary for the safety of the trial subject.

***7.8 Overview of the Safety Reporting Process/Pharmacoviligance responsibilities***

## The PI will have the overall pharmacovigilance oversight responsibility. The procedure for ensuring that all SAE/SUSAR Reporting is conducted in accordance with the Sponsor’s timelines is illustrated in the flow diagram in Appendix G

## Each participant will receive a wallet-sized Trial ID card giving contact details in case of questions relating to the trial (Appendix F)

**8. STATISTICAL CONSIDERATIONS**

***8.1 Number of Participants***

The Polypill is expected to reduce total cholesterol by about 1.8mmol/L and blood pressure by 20mmHg and 11mmHg (systolic and diastolic respectively). To be conservative, the power calculations were based on there being reductions of 1.3 mmo/L of serum cholesterol and 18mmHg systolic and 9 mmHg diastolic blood pressure. A trial of 100 participants would have over 90% power to detect these changes at the 5% level of significance.

***8.2. Data Analysis***

A statistical analysis of the results will be undertaken at the completion of the study. The placebo adjusted differences in serum cholesterol and systolic and diastolic blood pressure will be computed. The proportion of participants reporting adverse effects on Polypill and placebo will be compared and placebo adjusted excess incidence of adverse effects calculated. The placebo-adjusted differences in pill counts at the end of each 3 month period will be calculated together with adherence to the Polypill after 3 and 6 months.

***8.3. Data access and security***

All records will be kept securely and confidentially and analyses for reports and publications will present anonymous details. The Wolfson Institute of Preventive Medicine is part of Barts and The London Queen Mary’s School of Medicine, University of London which is registered under the Data Protection Act. In the Wolfson Institute there are two levels of security: (i) the data will be coded and kept on a network fileserver to which access is available only to a limited number of authorised users; (ii) user permissions ensure that even when logged on to the server the database is invisible to all but specifically authorised users. The server is behind a “firewall” which isolates it from the rest of the College network. It is physically protected in a locked room to which only computer staff have access, backed up every evening. Access to the building is controlled.

**9. Data Handling & Record Keeping**

***9.1 Confidentiality***

The Investigators will ensure that patient anonymity is protected and maintained. They will ensure that their identities are protected from any unauthorised parties. Information with regards to study patients will be kept confidential and managed in accordance with the Data Protection Act, NHS Caldicott Guardian, The Research Governance Framework for Health and Social Care and Research Ethics Committee Approval. All participant identifiable information will be anonymised with regard to any report or publication relating to this study.

The same standard will be followed if any patient information needs to be sent to a third party (including correspondence/communication to central laboratories, CROs, or sponsor).

**9.2 *Case Report Form***

The Study investigators will be responsible for completion of the case Report Form for each participant which will collect information as detailed in section 5.5 and 5.6 and any SAE’s that are reported.

***9.3 Record Retention and Archiving***

All records are the responsibility of the Investigators and will be kept in secure conditions. When the research trial is complete, it is the intention that the records will be kept for a further 20 years.

***9.4 Compliance***

The Investigators will ensure that the trial is conducted in compliance, the principles of GCP and the Royal College of Physicians Guidance on Research Ethics and in accordance with all applicable regulatory requirements including but not limited to the Research Governance Framework and the Medicines for Human Use (Clinical Trial) Regulations 2004, as amended in 2006 and 2008, Trust and Research Office policies and procedures and any subsequent amendments.

***9.5 Clinical Governance Issues***

*Ethical Considerations*

This protocol and any subsequent amendments, along with any accompanying material provided to the patient in addition to any advertising material will be submitted by the Investigator to an Independent Research Ethics Committee. Written Approval from the Committee will be obtained and subsequently submitted to the JRO to obtain Final R&D approval.

***9.6 Quality Control and Quality Assurance***

The Principal nvestigator will be responsible for reporting any serious breaches of the Protocol to the sponsor (JRO) within 24 hours of hearing of such events. The sponsor will notify and report to the MHRA within 7 working days of becoming aware of the serious breach.

**10. Trial Committees**

A data monitoring committee will be convened after the end of the 6 month cross-over trial to review the results on risk factor reduction and reported adverse effects. They will determine if, on the basis of the trial results or any other information available, there are any reasons to discontinue the 2 year monitoring phase of the study.

**11. Publication Policy**

It is our intention to present the results of the cross-over study at scientific meetings and publish the results in scientific journals. All data will be anonymised for this purpose.

**12. INSURANCE TO COVER INDEMNITY FOR NON-NEGLIGENT HARM**

Queen Mary (University of London) will provide indemnity for non-negligent harm and will provide indemnity for its staff for negligent harm. Compensation for any injury caused by taking part in this study, however unlikely, will be in accordance with the guidelines of the ABPI. These guidelines recommend compensation without the participant having to prove that the investigators are at fault. This applies in cases where it is likely that such injury results from the participant following this protocol. The participant’s right at law to claim compensation for injury where he or she can prove negligence is not affected.

**13. REFERENCES**

| 1 | a | Wald NJ, Law MR. A strategy to reduce cardiovascular disease by more than 80%. *BMJ* 2003;**326**:1419-23 |
| --- | --- | --- |
| 2 | b | Law MR, Wald NJ, Rudnicka AR. Quantifying effect of statins on low density lipoprotein cholesterol, ischaemic heart disease and stroke: systematic review and meta-analysis. *BMJ* 2003;**326**:1423-7 |
| 3 | c | Law MR, Wald NJ, Morris JK, Jordan RE. Value of low dose combination treatment with blood pressure lowering drugs: analysis of 354 randomised trials. *BMJ* 2003;**326**:1427-31 |
| 4 | f | Law MR, Wald NJ. Risk factor thresholds: their existence under scrutiny. *BMJ* 2002;**324**:1570-6 |
| 5 | d | Prospective Studies Collaboration. Age-specific relevance of usual blood pressure to vascular mortality: a meta-analysis of individual data for one million adults in 61 prospective studies. *Lancet* 2002;**360**:1903-13 |
| 6 | l | Asia Pacific Cohort Studies Collaboration. Blood pressure and cardiovascular disease in the Asia Pacific region. *J Hypertens* 2003:**21**:707-16 |
| 7 | m | MacMahon S, Peto R, Cutler J, Collins R, Sorlie P, Neaton J, et al. Blood pressure, stroke and coronary heart disease: part 1, prolonged differences in blood pressure: prospective observational studies corrected for the regression dilution bias. *Lancet* 1990;**335**:765-74 |
| 8 | g | Collins R, Peto R, MacMahon S, Hebert P, Fiebach NH, Eberlein KA, et al. Blood pressure, stroke and coronary heart disease: part 2, short-term reductions in blood pressure: overview of randomised drug trials in their epidemiological context. *Lancet* 1990;**335**:827-38 |
| 9 | n | Law M, Wald N, Morris J. Lowering blood pressure to prevent myocardial infarction and strokes: a new preventive strategy. Health Technology Assessment 2003;Vol 7,No 31 |
| 10 | e | Law MR, Wald NJ, Thompson SG. By how much and how quickly does reduction in serum cholesterol concentration lower risk of ischaemic heart disease? *BMJ* 1994;**308**:367-72 |
| 11 | r | Cholesterol Treatment Trialists’ Collaborators. Efficacy and safety of cholesterol-lowering treatment: prospective meta-analysis of data from 90 056 participants in 14 randomised trials of statins. *Lancet* 2005;**366**:1267-78 |
| 12 | p | Wald NJ, Hackshaw AK, Frost CD. When can a risk factor be used as a worthwhile screening test? *BMJ* 1999;**319**:1562-5 |
| 13 | z | Wald DS, Wald NJ The polypill in the primary prevention of cardiovascular disease. Fundam Clin Pharmacol 2010;24:29-35 |

**Appendix A: Sample information for IMP Label**

**PPT 1 Trial**

Polypill Prevention Trial 1

For Clinical Trial Use

A study being conducted at the Wolfson Institute of Preventive Medicine, Barts and the London School of Medicine, Queen Mary University of London

**Participant Number : 01** **Period 1 tablets**

Contains 100 tablets of:

Polypill (simvastatin 40mg, hydrochlorothiazide 12.5mg, losartan 25mg, amlodipine 2.5mg) or placebo

Take one tablet each evening

**Helpline: 0207 882 6289**

Batch Number xxxx Expiry date: [……..]

Keep out of sight and reach of children. Store at room temperature

**Appendix B: Letter from Dr David Wald on WIPM letterhead
to participants of the Polypill Prevention Programme**

**The Polypill** **Prevention Trial**

***Preventing Heart Attack and Stroke***

**Dear [Name]**

I am writing to invite you to join a 6 month clinical trial on the Polypill, a 4-in-1 tablet designed to reduce a person’s risk of cardiovascular disease.

A Polypill has been made for the prevention of heart attacks and strokes in people who have not already had a cardiovascular disease event. It contains Simvastatin 40mg, Hydrochlorothiazide 12.5mg, Amlodipine 2.5 mg and Losartan 25mg in a single pill (shown below). We believe this is the best composition to give the greatest reduction in cholesterol and blood pressure with the lowest possibility of side-effects.

Simvastatin


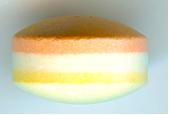


Losartan & hydrochlorthiazide

Amlodopine

**What does the study involve?**

Everyone in the study would have a period taking the Polypill and a period taking a dummy (placebo) pill, each period lasting 3 months and the sequence allocated at random. All the tablets used in the study look identical. The trial is double blind so that neither you nor the doctors providing the pills know which treatment is being taken at any time. We will measure cardiovascular risk factors (blood pressure and blood cholesterol) during the two periods so that we can determine the extent to which the Polypill influences these risk factors.

After completion of the two treatment periods everyone will be able to continue to receive the Polypill. You would be contacted periodically to check on any symptoms, to see whether you are remembering to take your Polypill each day and to find out if you wish to receive your next 3 month supply of Polypill, similar to the periodic contact you currently receive in the programme.

The attached information leaflet describes the study. People aged 50 and over who are currently receiving individual Polypill components as part of the Polypill Prevention Programme conducted at the Wolfson Institute of Preventive Medicine will be eligible for the trial. The Polypill would replace what you are currently taking.

Please take do not hesitate to ask any questions, by calling 0207 882 6298.

Yours sincerely,

David Wald

Version 1, 28 June 2010

**Appendix C: Information Leaflet**


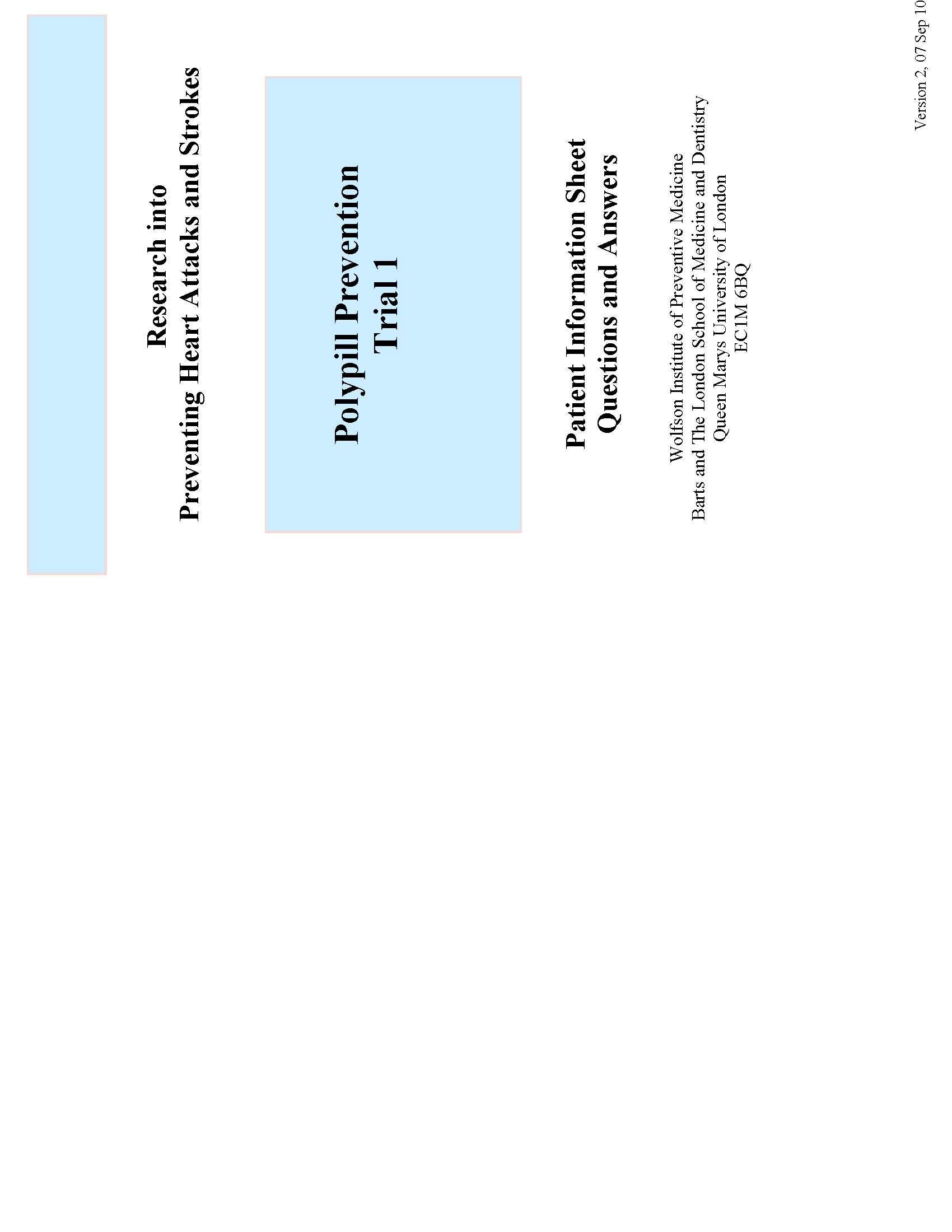


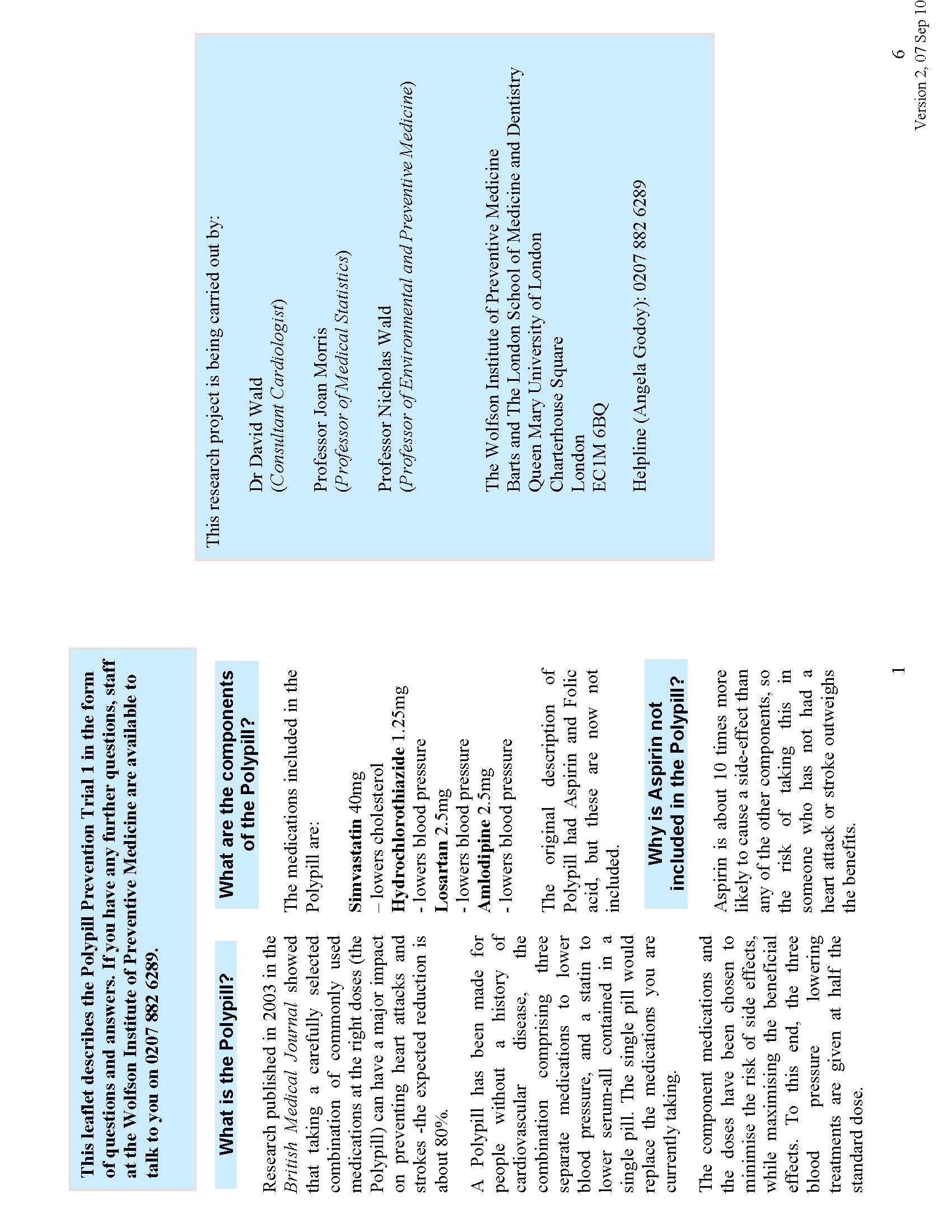


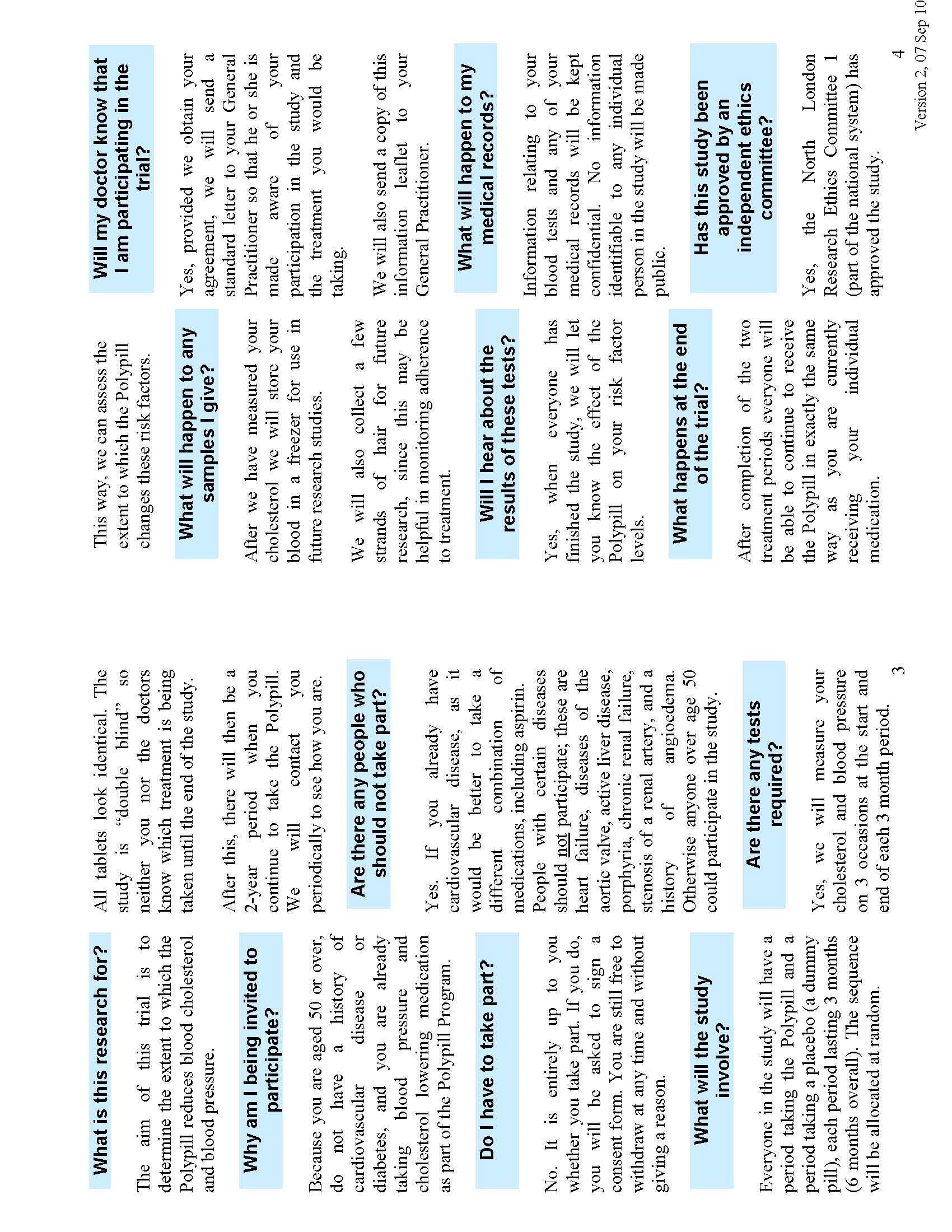


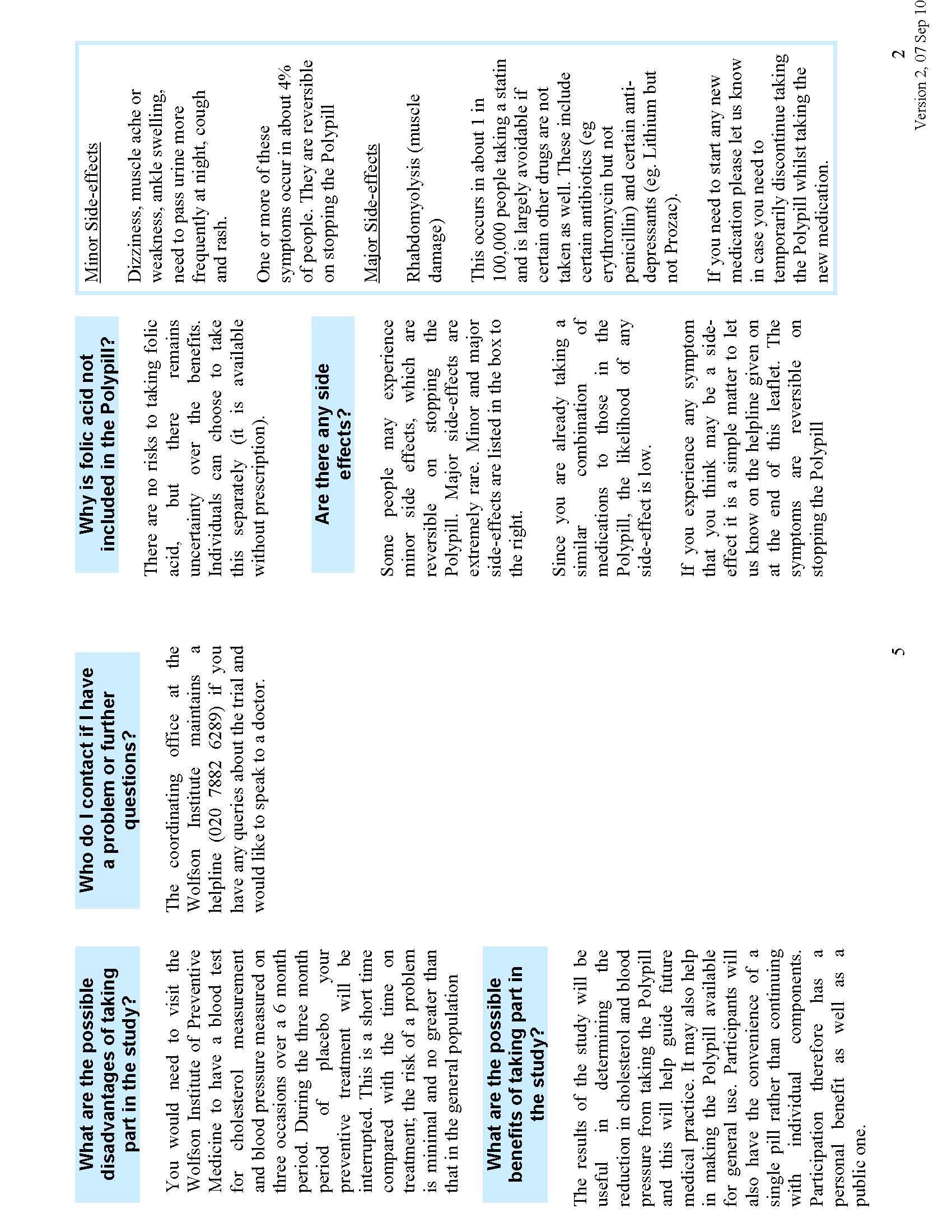


**Appendix D: Consent form**

Centre for Environmental and Preventive Medicine

| WOLFSON INSTITUTE OF PREVENTIVE MEDICINE  “Queen Mary & Westfield College, University of London” | Charterhouse Square London EC1M 6BQ |
| --- | --- |

Tel: +44 (0)20 7882 6190

Fax: +44 (0)20 7882 6270

Direct Line: +44 (0)20 7882 6298

E-mail: d.s.wald@qmul.ac.uk

Participant Identification Number:

Investigator:…………………………………………

**Polypill Prevention Trial 1**

**CONSENT FORM**

**Please initial box**

| 1. I confirm that I have read the information sheet dated 07 September 2010 (version 2), for the above study and have had the opportunity to ask questions. |  |
| --- | --- |
|  |
| 1. I understand that my participation is voluntary and that I am free to withdraw at any time, without giving any reason, without my medical care or legal rights being affected. |  |
|  |
| 1. I understand that relevant sections of my medical notes, data collected during the study and data provided by the NHS Information Centre, may be examined by responsible individuals from regulatory authorities and/or from Barts and the London/Queen Mary University of London, where it is relevant to my taking part in the study or in future research. I give my permission for these individuals to have access to my records. |  |
|  |
| 1. I agree that my blood samples can be stored in the Wolfson Institute of Preventive Medicine for future research. |  |
|  |
| 1. I agree to a sample of my hair can be collected for the measurement of drug metabolites and stored at the Wolfson Institute of Preventive Medicine |  |
|  |
| 1. I agree to take part in the above study |  |
|  |

________________________ ________________ ____________________

Name of participant Date Signature

________________________ ________________ ____________________

Name of Investigator Date Signature

One copy for researcher, one for participant

Version 2, 7 September 2010

**Appendix E: Letter from Dr David Wald to GPs on Wolfson Institute of Preventive Medicine letter head paper**

Dear Dr GP,

We are writing to let you know about a study we are conducting at Queen Mary & Westfield College, University of London (The Barts and the London, Queen Mary School of Medicine and Dentistry, The Wolfson Institute of Preventative Medicine) and to say that your patient Mr/Mrs………… has agreed to take part.

A single pill (Polypill) containing 4 components (simvastatin, amlodipine 2.5mg, hydrochlorothiazide 12.5mg and losartan 25mg) has been made for the prevention of heart disease and stroke in people without a history of theses disorders.

This study aims to assess the effect of the Polypill in reducing cholesterol and blood pressure among participants of the Polypll prevention Programme – people who are already taking blood pressure and cholesterol lowering treatment as individual component drugs under the supervision of the Wolfson Institute of Preventive Medicine.

The study is a randomized crossover trial in which each participant takes the Polypill and a placebo in random order. Each treatment period lasts 3 months. All the tablets used in the study are identical and the trial is double blind so neither we nor the participant knows which treatment is being taken at any one time. Once the trial is complete the code will be broken and the sequence revealed. At the completion of the trial, participants will receive the Polypill for a period of two years and any symptoms attributable to adverse effects will be recorded.

We have ensured there are no medical contraindications to Mr/Mrs ….. participating in the trial and made it clear that the decision to participate is entirely voluntary. We have informed potential participants that the side effects of the Polypill are uncommon and that, if at any stage, they think they are experiencing a side effect or have any query about the study they may call the Wolfson Institute of Preventive Medicine “helpline” (0207 882 6289).

I enclose a leaflet about the trial which has been sent to Mr/Mrs….. who may consult you before deciding whether to participate. Please do not hesitate to contact us if you wish to know anything further about the study.

Yours sincerely,

Dr David Wald

Senior Lecturer & Consultant Cardiologist

Version 1, 28 June 2010

**Appendix F**

# THREE MONTHLY SYMPTOM REPORTING QUESTIONNAIRE

**Polypill Prevention Trial**

Patient name:

Patient ID number:

Investigator:

Visit number:

Date:

Have you noticed any significant increase in the following symptoms over the past 3 months

**NO** YES **If Yes, sufficient to stop tablets?**

NO YES

Dizzinness

Ankle swelling

Flushing

Generalised Muscle aches

Generalised Muscle pain

Cough

Rash

Tongue or lip swelling

Have you experienced any other symptoms in the past month which you attribute to the medication?

NO YES

Describe:

**If Yes, sufficient to stop tablets?**

NO YES

Version 1, 28 June 2010

**Appendix G**

**SAE/SUSAR Reporting Flow Diagram**

Study Investigators at Wolfson Institute of Preventive Medicine learn of possible SAE/SUSAR

PI consider whether reported episode qualifies as SAE/SUSAR.

PI considers recording event as AR/AE in CRF

PI notifies JRO (Sponsor) within 24 hours of becoming aware of SAE/SUSAR

JRO/Sponsor reports SAE/SUSAR to MHRA within 7 days as per standard protocol

Reported to PI within 24 hours

NO

YES

# Appendix H

# Participant ID Card

Front of ID Card

***PPT01***

Note to patient: This card contains information on the composition of the Polypill which may be helpful for any doctor or healthcare professional needing to treat you: It also confirms that you are taking part in the Polypill Prevention Trial and gives contact details of the study team in case it is necessary to speak to a study doctor.

Randomisation Number:

Study Medication: Amlodipine 2.5mg, Simvastatin 40mg,

Losartan 25mg, Hydrochlorothiazide 12.5mg

Patient ID Card V1, 28 june 2010

Back of ID Card

***PPT01***

To the physician/healthcare professional:

This patient is taking part in a clinical study. In case of an emergency please contact the study team on the number below and you will be able to speak to a study doctor:

**Tel No: 02078826289**

**Address:** Charterhouse Square, Queen Mary University of London, EC1M 6BQ

# APPENDIX I

Documentation of minor corrections/clarifications arising from errors and omissions in this

document

**Page Correction/Clarification Date recorded**

12 Correction: (omitted) “of adherence” deleted from flow diagram 19/1/2012

13 Clarification that Entry not determined by BP or so, off-treatment 19/1/2012

(baseline) values not determined

18 Correction that pill counts will not be assessed at Visit 1 19/1/2012

19 Correction: “ At Randomisation” replaced “Baseline” in schedule 19/1/2012
